# DOES FIVE-STAR QUALITY RATING MATTER FOR NURSING HOME QUALITY DURING THE COVID-19 PANDEMIC?

**DOI:** 10.1093/geroni/igac059.1559

**Published:** 2022-12-20

**Authors:** Lei Yu, Xiao Qiu, Na Sun

**Affiliations:** Miami University, Oxford, Ohio, United States; Miami University, Oxford, Ohio, United States; Miami University, Oxford, Ohio, United States

## Abstract

Objectives Nursing homes (NH) confronted tremendous difficulties considering confirmed residents Covid-19 cases and deaths in the U.S. The Centers for Medicare & Medicaid Services (CMS) applies the Five-Star Quality Rating (FSQR) to indicate the quality of care in nursing homes based on health inspection surveys, staffing as well as care process and resident outcomes during the COVID-19 pandemic. This study aims to examine whether FSQR was related to the total number of NH resident Covid-19 cases and deaths.Design This study analyzed 6,978 nursing homes across the country with data from CMS Nursing Home Compare, CMS COVID-19 Nursing Home Public File, Long-term Care Focus, Payroll Based Journal, Rural-Urban Commuting Area. Negative binomial regressions were used to investigate associations between FSQR and NH COIVD-19 outcomes controlling for state fixed effects and clustering of nursing homes within counties. The characteristics of facility, residents, payer-mix, nursing staff, and geographic location were also controlled. ResultsComparing to NH with 1-star in Health Inspection, Staffing, or Overall ratings, NH with better performance have lower risk of having increased number of COVID cases and deaths among residents. Further, nursing home Quality Measures rating is not significantly associated with residents’ COVID-19 deaths. ConclusionOverall, the FSQR is a useful measure of quality in part when investigating NH’s performance during the COVID-19 pandemic. Future policymakers should pay special attention to providers performing poorly in FSQR when improving the quality of nursing homes, particularly regarding infection control.

